# Die Erteilung der Venia legendi als Gradmesser einer einsetzenden Fachdifferenzierung: Victor von Ivánchich de Margita (1812–1892) „Docent für die Chirurgie der Harnorgane“ in Wien, 1851*

**DOI:** 10.1007/s00120-022-01904-6

**Published:** 2022-08-09

**Authors:** Friedrich H. Moll, Thorsten Halling, Shahrokh F. Shariat

**Affiliations:** 1 Institut für Geschichte, Theorie und Ethik der Medizin, Centre for Health and Society, Medizinische Fakultät, Heinrich-Heine-Universität, Düsseldorf, Deutschland; 2 Urologische Klinik, Kliniken der Stadt Köln gGmbH, Neufelder Straße 32, 51067 Köln, Deutschland; 3 Curator Deutsche Gesellschaft für Urologie e. V, Düsseldorf-Berlin, Deutschland; 4 Department of Urology, Comprehensive Cancer Center, Medical University Vienna, Vienna General Hospital, Vienna, Österreich

**Keywords:** Habilitation, Fachdifferenzierung Urologie, Fachspezialisierung, Geschichte der Medizin, Geschichte der Urologie, Wiener Medizinische Schule, Habilitation, Urology as a specialty, Networking urology, History of medicine, History of urology, Vienna School of Medicine

## Abstract

Neben Paris gehört Wien zu den frühen Zentren der sich im 19. Jahrhundert differenzierenden und spezialisierenden Urologie. Gerade die II. Wiener Medizinische Schule (Erna Lesky) mit ihren Hauptvertretern Carl Freiherr von Rokitansky (tschechisch: Karel Rokytanský; 1804–1878) und Joseph Ritter von Škoda (1805–1881) bot das wissenschaftliche Umfeld für Studierende und Ärzte, sich mit neuen wissenschaftlichen Methoden wie klinische Chemie, Labormedizin und Mikroskopie bekannt zu machen. Am Beispiel eines frühen Urologen wollen wir den Habilitationsvorgang nachzeichnen und einordnen.

## Abstract (en)

Alongside Paris, Vienna was one of the early centers of specialization and professionalization in medicine and urology in the 19th century. Especially the 2nd Vienna Medical School (Erna Lesky) with its main representatives Carl Freiherr von Rokitansky (in Czech: Karel Rokytanský; 1804–1878) and Joseph Ritter von Škoda (1895–1881) was able to create the perfect scientific environment for young students to become acquainted with new fields of research often in an interdisciplinary setting, e.g., chemistry, microscopy or pathology in combination with clinical departments like surgery. We analyze the process of habilitation using the example of a urologist to outline this process within the history of science.

## Einführung

Bekanntermaßen begann die Fachdifferenzierung und Herausbildung einer Spezialdisziplin wie die der Urologie unter den Kriterien der naturwissenschaftlichen Medizin ab dem ersten Drittel des 19. Jahrhunderts in den europäischen Zentren der Medizin, Paris, Wien, Berlin und London, geprägt durch die jeweiligen lokalen und nationalen Bedingungen der jeweiligen Hochschulmedizin. Nicht jede Aufspaltung in der Medizin führte jedoch zur Entstehung und Verfestigung einer eigenen Disziplin. Untersuchungen hierzu stellen bis heute ein Desiderium dar, insbesondere, da hier wissenschaftssoziologische, wissenschaftshistorische und wissenschaftspolitische Fragen aufgeworfen werden [1–4].

Einige Autoren sehen den Begriff „Urologie“ für die Bezeichnung des weiteren Faches ohne Engführung auf die „Harnschau“ erst ab dem Ende des 19. Jahrhunderts.

Die institutionellen Strukturen von Hochschulen sehen hier Verfahren zur Einrichtung vor, die als Gradmesser der erreichten Fachetablierung angesehen werden können:

- *Promotionen*, die unter dem neuen Fach angenommen werden;
- *Habilitationen *(Erteilung der Lehrbefähigung: Facultas docendi) und der Venia legendi (Erteilung der Lehrberechtigung), die den neuen Fachbegriff enthalten;
- *Dozenten*, Lehrbeauftragte oder apl. Professoren von außerhalb der Universität, die sich als Spezialist für das neue Fach bezeichnen;
- *Extraordinariate* unter dem neuen Fachbegriff;
- *Ordinariate* [6].

Daher fragen wir exemplarisch nach den universitären Rahmenbedingungen und Kriterien für die Abgabe einer Lehrqualifikation in einer neuen, sich entwickelnden medizinischen Disziplin und nach der Bedeutung für das Fach, die dieser Prozess an einem ausgesuchten Wissenschaftsstandort bedeutete. Dies streift auch Aspekte der Universitätsgeschichte, die kein esoterisches Gebiet der Ideengeschichte mehr ist [7, 8].

## Was definiert eine eigene wissenschaftliche Disziplin?

Die Urologie ist in ihrer Eigenwahrnehmung ein Querschnittsfach mit der Konzentration auf ein Organsystem – den Harntrakt und die männlichen Geschlechtsorgane. Das Fach setzt sich gleichrangig aus Anteilen der operativen Medizin und der konservativen Medizin zusammen und zeichnet sich durch einen überproportionalen Einsatz von endoskopischen und minimal-invasiven Techniken aus. Schon seit seiner Etablierung im Rahmen der naturwissenschaftlichen Medizin im ersten Drittel des 19. Jahrhunderts in Paris bestehen Abgrenzungen insbesondere zu weiteren sich generalistisch auffassenden operativen Fächern wie der Chirurgie oder der Frauenheilkunde. Zuvor übten die handwerklich ausgebildeten Lithotomisten ihren Beruf lange im Umherziehen aus, während Wundärzte, Bader sowie Feldscherer/Regimentschirurgen in Friedenszeiten zumeist ortsansässig waren.

Die akademische Disziplinenbildung ist ein Vorgang funktionaler Differenzierung, an dem das Wachstum des Wissens, der Forschungs- und Lehrstätten sowie Erfordernisse der Arbeitsteilung ursächlich beteiligt sind. Die akademische Fächerstruktur entstand in eigendynamischen Wachstumsprozessen, die ganz wesentlich von dem Autonomiestreben der beteiligten Akteure geleitet waren [9, 10]. Die Entstehung und Entwicklung neuer Disziplinen folgte nur selten dem von dem Wissenschaftsphilosophen Thomas Kuhn (1922–1996) beschriebenen Muster revolutionärer Umbrüche im Wissenschaftssystem [11, 12], sondern vielmehr einem evolutionären Modell der allmählichen Ausdifferenzierung [13] und Verselbstständigung neuer Forschungsrichtungen [14].

## Zur Entwicklung und Differenzierung der Urologie im „langen“^1^ 19. Jahrhundert

Das Fachgebiet der Urologie entwickelte sich im 19. Jahrhundert – ähnlich der Chirurgie – mit dem Erstarken des naturwissenschaftlichen Paradigmas von einer handwerklich orientierten Disziplin der vornaturwissenschaftlichen Ära, die durchaus Wurzeln bei den handwerkschirurgischen Steinschneidern hatte, zu einer naturwissenschaftlichen, früher als die Chirurgie auf einen funktionsorientierten Blick ausgerichteten, technisch hoch affinen medizinischen Spezialdisziplin, die u. a. neben der Chirurgie auch mit der erstarkenden Frauenheilkunde, aber auch der medizinischen Klinik und der Sexologie sowie der Venerologie enge Berührungspunkte aufwies. Die These, dass die Disziplin der Urologie eine reine Abspaltung aus einer im 19. Jahrhundert nun lokalistisch ausgerichteten Chirurgie ist, wurde nicht nur von Chirurgen [16, 17], sondern auch von Medizinhistorikern [18–20] repetiert und publiziert. Dies ist jedoch eher als ein aus der chirurgischen Fachabgrenzung besonders nach Einführung des Facharztstatus 1924 („Bremer Richtlinie“) im Deutschen Reich [21–24] oder der Regulierungen in Österreich ab 1935 [25] heraus zu verstehendes Erzähl- und Selbstvergewisserungsmuster einer sich weiter differenzierenden operativen Medizin/Chirurgie zu deuten. Während mittlerweile eine vielfältige Literatur zur Entwicklung der Wundärzte [26, 27], zur allgemeinen Fachspezialisierung oder Professionalisierung in Medizin und Naturwissenschaften [28] und zu Fachkulturen vorliegt, fehlen noch immer wissenschaftshistorische und epistemiologische exemplarische Untersuchungen zur Urologie und deren Fachdifferenzierung im 19. Jahrhundert, besonders im Hochschulbereich der frühen medizinischen Fachzentren Wien, Berlin, London, Paris [29, 30].

Eine Bedingung zur Entstehung neuer Disziplinen in den Naturwissenschaften ist oft eine technische Neuheit, die ein technisches Problem löst. Diese Entwicklungen laufen häufig außerhalb von Universitäten ab.

Nach kommunikativer Etablierung in wissenschaftlichen Netzwerken und Publikationen wie Fachzeitschriften und Büchern entwickeln sich aus der entstehenden „scientific community“ neue Berufsfelder und akademische Laufbahnen („disziplinäre Professionalisierung“), die die Institutionalisierung vorantreiben und mit ihrer Professionalisierungspolitik die Verstetigung der neuen Disziplin fördern [31]. Die Entwicklung auch im Bereich der Urologie verlief in Phasen und Wellen [32]. Hier zeigen sich jedoch deutliche nationale Unterschiede sowohl in Bezug auf die Etablierung an Hochschulen durch Habilitationen wie auch durch Gründung wissenschaftlicher Fachgesellschaften.

## Frühe Habilitationen im Fachgebiet Urologie

Für die preußische Friedrich-Wilhelms-Universität in Berlin lassen sich mit der Habilitation von Max Nitze (1848–1906) in Chirurgie [33] sowie Carl Posner (1854–1928) in Innerer Medizin [34] 1889 erste Privatdozenturen, die sich auf urologische Themenstellungen gründeten, in den benachbarten Fachbereichen wie Chirurgie bzw. Innerer Medizin nachweisen, ohne den Begriff Urologie zu führen. Die erste Habilitation rein für das Fachgebiet Urologie erfolgte in Berlin erst 1942 mit dem NSDAP-Mitglied Karl Heusch (1894–1986^2^; [36–38]).

Der Zugang zur Privatdozentur und die damit einhergehenden Rechte und Pflichten waren an der Berliner Universität erstmals im deutschen Sprachraum im Jahre 1816 festgelegt worden, zuvor wurde noch der Begriff „des zur Vorlesung Berechtigten“ vewandt [39]. Andere deutsche Universitäten folgten diesem Beispiel, sodass die Institutionalisierung von Habilitation und Privatdozentur im deutschsprachigen Raum ab etwa 1850 als abgeschlossen gelten kann [40, 41].

In Wien wurde die Habilitation als akademisches Verfahren 1848/1849 im Rahmen der Thun’schen Universitätsreform fest eingeführt (Habilitationsordnung für Privatdozenten) und im ersten Jahr 18 Habilitationen an der Medizinischen Fakultät durchgeführt [42–46]. Die Habilitation wurde als „*Garantie … für die Brauchbarkeit der Privatdozenten*“ angesehen, denn die Prüfung der Fähigkeiten erfolgte durch den Lehrkörper und später das Professorenkollegium

„*dem sie aggregiert und dessen Mitgliedern sie zum Teil Concurrenz machen werden, bei dem also keine Motive zur laxen Behandlung dieser Prüfung vorauszusetzen“* waren [47].

Die Habilitation erfolgte in Wien stets für ein konkretes Fach, welches mit der Fakultät, an der sich der Bewerber habilitieren wollte, kompatibel sein musste [48]. Die Ordnung von 1848 schwieg sich zum Fachumfang aus. Die Ordnung von 1888 legte fest, dass nur für den ganzen Umfang einer Disziplin oder einen größeren Bereich, der als abgeschlossenes Ganzes gesehen werden konnte, die Habilitation erfolgen konnte [49].

Eine weitere Habilitation für das Fach „Urologie“ erfolgte in Wien mit Viktor Blum (1877–1954) im Jahre 1912 [50]. Vorher hatte sich Leopold von Dittel (1815–1898), der sich de facto überwiegend urologisch betätigte, im Fach „Operative Chirurgie“ 1856 mit einem chirurgisch-orthopädischen Thema habilitiert [51] (a. o. Professor 1865). Robert Ultzmann (1842–1889), der später an der Allgemeinen Poliklinik tätig war, habilitierte sich im Jahre 1872 für das Fach „Erkrankungen der Harnorgane“ [52], Gustav Jurié Edler von Lavandal (1842–1924)^3^ 1874 für die „Chirurgie der Harn- und Geschlechtsorgane“^4^.

Das Wissen um die frühe „Habilitation für Urologie“ von Viktor von Ivánchich ist in der Wiener urologischen Erinnerungskultur wie auch von der von Robert Ultzmann für „Erkrankungen der Harnorgane“ oder Gustav Juriés verloren gegangen^5^, da in der späteren Literatur Viktor Blum als „erster Habilitant“ für Österreich und Deutschland aufgeführt wurde [56]. Dies kann sogar auf Viktor Blum selbst zurückgegangen sein, der in seinem sehr ausführlichen Festvortrag 1929 zur „Geschichtlichen Bedeutung der Wiener Urologie“ Viktor von Ivánchich und Gustav Jurié nicht erwähnte [57], während noch 1884 der Medizinhistoriker Theodor Puschmann (1844–1899) zur Hundertjahrfeier des Allgemeinen Krankenhauses in Wien, also zu Lebzeiten von Ivánchichs, Ultzmanns und Juriés, diese drei Protagonisten in einer Übersicht „Docenten“ mit ihrem Habilitationsfach aufführte [58].

**Figure 1:**
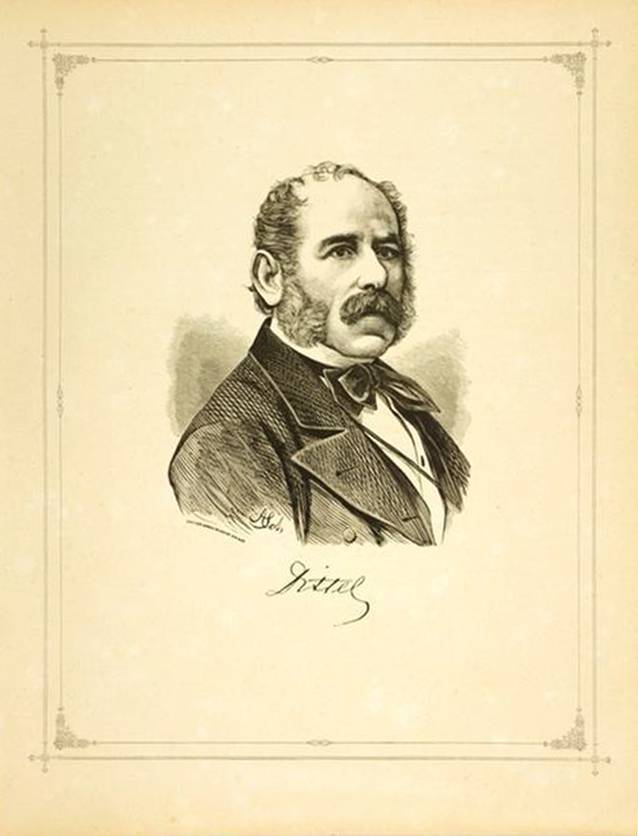

**Figure 2:**
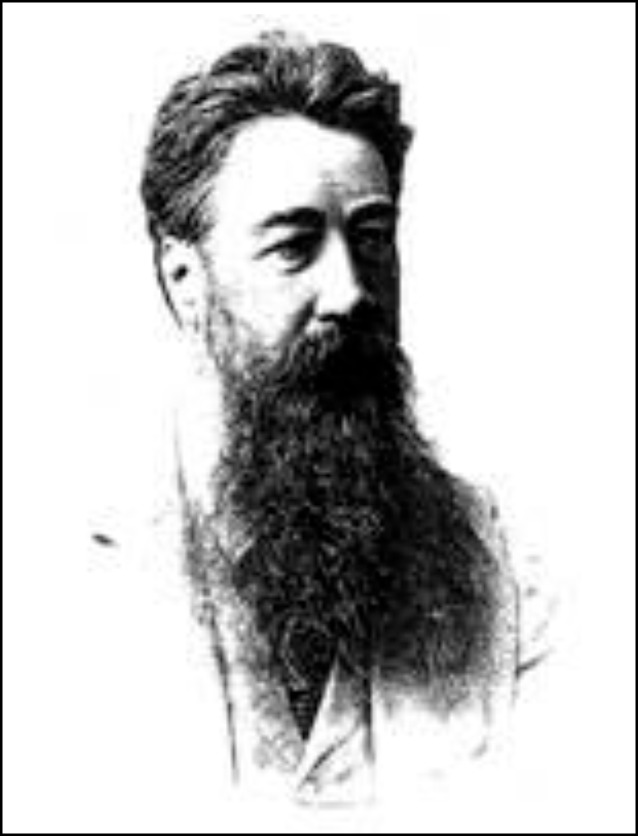

**Figure 3:**
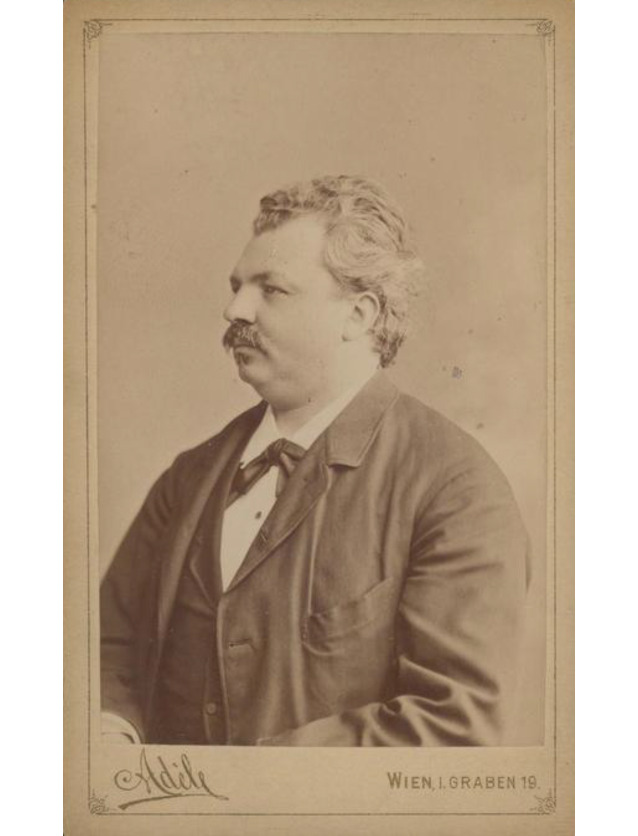

**Figure 4:**
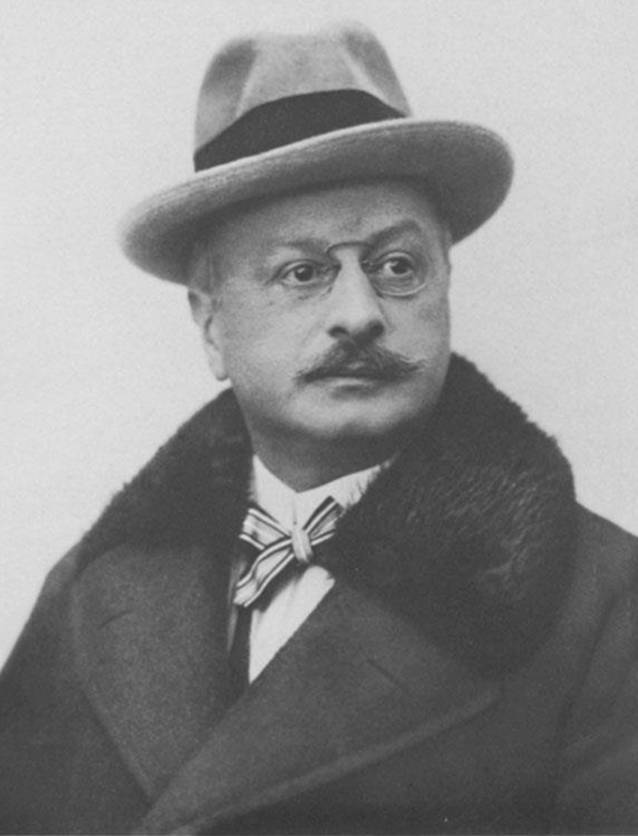

Bereits 1911 tauchte von Ivánchichs Name nicht mehr in einer sehr ausführlichen Arbeit eines unbekannten Verfassers „Zur Geschichte der Urologie in Wien“ [59] auf, während Robert Ultzmann mit seinen wissenschaftlichen Ergebnissen sowie einer großformatigen Portraitfotografie als Blickfang des Artikels besonders gewürdigt wurde (Abb. 1, Dittel, Abb. 2 Jurié, Abb. 3, Ultzmann, Abb. 4, Blum).

In Leipzig (Königreich Sachsen) hatte sich Arthur Kollmann (1858–1941) – eine Nennung des Fachgebiets erfolgte im Aktenlauf im Gegensatz zu Wien nicht – über ein hämatologisches Thema („Beiträge zur Pathologie und pathologischen Histologie des Blutes“), das von dem Internisten Heinrich Curschmann (1846–1910) begutachtet wurde und dem Probevortrag „Die neueren physikalisch-diagnostischen Methoden bei Erkrankungen der Blase und der Harnröhre“ – am 01.08.1890 habilitiert. Kollmann hielt nur Vorlesungen über urologische Themen [61, 62].

An der Münchener Ludwig-Maximilians-Universität gelang Ludwig Kielleuthner (1876–1972) 1914 die Habilitation im Fach „Urologie“ [63, 64].

In Düsseldorf habilitierte sich Paul Janssen (1874–1974) 1910 für „Chirurgie und Urologie“, eine Kombination, die bis in die 1970er-Jahre in der Bundesrepublik Deutschland die integralistische Haltung von Chirurgen in Hochschulgremien deutlich widerspiegelte.

In Köln wurde Gottfried Thelen (1871–1941) im Jahre 1912 „nur“ zum „Dozenten für Cystoskopie“ an der „Akademie für Praktische Medizin“ im Jahre durch den Preußischen „Minister der Geistlichen‑, Kultus- und Medicinalangelegenheiten“ nach Einreichung von Publikationen ernannt [65]. Mit der Ernennung war nicht die Gestellung von Betten für den klinischen Unterricht oder eine Bezahlung verbunden.

In Wien konnte sich der in Budapest geborene Victor von Ivánchich bereits wenige Jahre nach Etablierung der Habilitation als universitärem Qualifikationsnachweis 1848 im Jahre 1851 in einem akademischen Prozess die „Venia legendi“ ohne Einreichung einer selbständigen Schrift, allein unter Anlage vorhandener Publikationen für „Urologie“, erlangen. Darüber hinaus betreute er am Allgemeinen Krankenhaus Betten, d. h. Patienten – ein Status, den auch in späterer Zeit viele Privatdozenten in Deutschland und Österreich nicht besaßen (Nitze, Posner, Casper in Berlin). Diese mussten für ihre Vorlesung Patienten aus ihrer Sprechstunde/Praxis requirieren.

Wissenschaftlich mit der „modernen“ innovativen, minimal-invasiven „blinden“ Blasensteinlithotripsie sowie mit der Urethrotomie zur Therapie gonorrhoischer Harnröhrenstrikturen hervorgetreten, gehörte Ivánchich somit zu den frühen habilitierten Protagonisten und akademischen Promotoren des sich gerade differenzierenden Spezialgebiets.

Unsere These ist, dass sich gerade zu Zeiten universitärer Reformen wie in Wien 1848 die Erteilung der „Venia legendi“ in einem „neuen“ Fach leichter durchsetzen ließen und nationale und lokale wissenschaftspolitische Entwicklungen hierbei eine entscheidende Rolle spielten [66–70]. Zwischen den frühen Habilitationen und der Etablierung der entsprechenden Lehrstühle („Lehrkanzeln“/Ordinariate) wie auch selbständiger Kliniken sollte dann im Bereich der Urologie noch eine längere Zeitspanne liegen.

In Wien wurde beispielsweise früh eine Urologischen Abteilung und Ambulanz innerhalb der II. Chirurgischen Klinik 1910 unter Julius Hochenegg (1859–1940) etabliert, eine eigene „Lehrkanzel für Urologie“ (aber) erst „1967(!)“ eingerichtet, nachdem im Jahre 1962 noch unter dem Chirurgen Leopold Schönbauer (1888–1963) eine „Urologische Universitätsklinik im Allgemeinen Krankenhaus“ unter Leitung des Urologen Richard Übelhör (1901–1977)^6^ als a. o. (!) Professor [71] etabliert worden war. Erst im Jahre 1967 wurde Übelhör dann zum ordentlichen Professor (Ordinarius, einer C4-Professur nach bundesdeutscher Regelung zu dieser Zeit vergleichbar) „erhoben“ und 1971 emeritiert (Tab. 1).

**Table 1:** 

Ort	1. Habilitationen	Etablierung Lehrstuhl
Wien	1851 v. Ivánchich, Urologie	1967 Übelhör (Habil 1936/1937)
	1856 v. Dittel, operative Chirurgie	
	1871 Josef Englisch, Chirurgie Thema der Antrittsvorlesung Urologie	
	1872 Ultzmann, Krankheiten der Harnorgane	
	1874 Gustav Jurié von Lavandal Chirurgie der Harn- und Geschlechtsorgane	
	1912 Blum, Urologie	
	1915 Paschkis, Urologie	
Berlin	1889 Nitze, Chirurgie	1966/1969 Brosig, FU 1977 Mebel, Charité (Dissertation B/Habilitation 1964)
	1889 Posner, Innere Medizin	
	1892 Casper, Chirurgie	
	1942 Heusch, Urologie	
Leipzig	1890 Kollmann, Krankheiten der Harnwege	1974 Dieterich (1970 Dissertation B/Habilitation)
Homburg/Saar	1948 Alken, Urologie (Sorbonne, Paris)	1947/48 Alken Homburg/Saar
Halle	1950 Stolze, Urologie	1958 Stolze Klinik Weidenplan
Jena	1958 Hientsch	1963 Hientsch
Köln	1906 Thelen, Cystoskopie	1972 Engelking
Düsseldorf	1910 Janssen, Chirurgie und Urologie	1963 Dettmar (Habil 1950 Chirurgie und Urologie)
	1950 Dettmar Chirurgie und Urologie	
München	1914 Kielleuthner, Urologie	1976 Schmiedt (Habil 1960)

### Zum Stand der Forschung

Interessanterweise ist Viktor von Ivánchich bisher nur ungenügend Ziel urologiegeschichtlicher Untersuchungen gewesen. Daher ist sein Name im Traditionskanon früher Urologen bzw. Proto-Urologen des deutschen Sprachgebiets oder auch Untersuchungen mit Fokus zur Urologie in Österreich nicht fest verankert. Im deutschen Sprachraum befassten sich nur die ehemaligen Archivare der DGU Johannes Keller (1899–1970; [72]) und eine darauf aufbauende Arbeit von Fritz Schultze-Seemann (1916–1987) damit. Dirk Schultheiss/B. Panning [73, 74] untersuchten Ivánchich unter dem Aspekt der Anästhesie in der frühen Wiener Urologie. Peter Paul Figdor (1926–2020), Archivar der ÖGU, führte Victor von Ivánchich in der „Biographie österreichischer Urologen“ auf [75]. Zu ihm existiert kein Eintrag im Österreichischen Bibliographischen Lexikon (ÖBL) 1815–1950 [76], aber ein Namenseintrag im Portal „Physicus“ der Wiener Universitätsbibliothek [77] und dem alten „Biographischen Lexikon des Kaiserthums Österreich“ [78]. Darüber hinaus ließen sich zwei ungarische Arbeiten nachweisen [79, 80].

Zu der technischen Entwicklung der „blinden“ inneren Urethrotomie sowie der „blinden Blasensteinlithotripsie“, den beiden hauptsächlichen Betätigungsfeldern Viktor von Ivánchichs, liegen im urologie- [81, 82] sowie medizinhistorischen [83] Schrifttum eine Vielzahl von Publikationen und historischen Untersuchungen seit deren Etablierung vor, auch mit lokalem Wiener Bezug [84, 85], wahrscheinlich um Primärautorenschaften [86] und lokale Wiener Besonderheiten und Prioritätsansprüche früh und nachhaltig zu dokumentieren und zu etablieren und diese auch in die jeweiligen Schulen und Erinnerungskulturen einzuordnen oder zu festigen [87–90]. Wissenschaftshistorische Untersuchungen zum Thema Habilitation als Exzellenzkriterium in der Urologie fehlen bisher. Der genaue akademische Ablauf wird bei den bioergographischen Untersuchungen aufgrund anderer Fragestellungen oder fehlender Quellen meist nur gestreift.

## Lebensweg Victor von Ivánchichs

Viktor von Ivánchich de Margita wurde im Jahre 20.02.1812 [91] in Pest als Sohn eines „städtischen Oberbeamten“ geboren. Die Einheitsgemeinde Budapest entstand erst im Jahre 1873 durch die Zusammenlegung der zuvor selbstständigen Städte Buda (dt. Ofen), Óbuda (Alt-Ofen), beide westlich der Donau sowie Pest östlich der Donau. Der Name Budapest selbst tauchte zuvor nicht auf, üblich im Sprachgebrauch war „Pest-Buda“. Ivánchich studierte dort Medizin und war als Assistent am dortigen St. Rochuspital tätig. Somit kann er zur aufstiegsbereiten Mittelschicht der Habsburger Monarchie gerechnet werden. Er promovierte in Pest 1834 zum Dr. med. („De musica medicae considerata“; [92]) und 1836 zum Dr. chir. [93].

Zwischen 1834–1836 weilte er zeittypisch zu einem Studienaufenthalt in Paris, wo die Fachverselbständigung und Differenzierung der Urologie bereits an der Wende zum 19. Jahrhundert eingesetzt hatte. Er hospitierte bei den wichtigsten Protagonisten der „blinden“ Blasensteinlithotripsie wie Jean Civiale (1792–1867; [94]), Leroy des Etiolles (1798–1860; [95]) Baron Charles Louis Stanislas Heurteloup (1793–1864; [96, 97]) sowie Jean Amussat (1796–1865). Hier wird von Ivánchich lebhaft die Diskussion um die klinische Überlegenheit der einzelnen Modifikationen, die sich in Paris um einen Prioritätsstreit erweiterte und die weite Wellen bis in den deutschsprachigen Bereich schlugen [98, 99], tagesaktuell verfolgt haben. Dies zeigen spätere Publikationen und Zeitschriftenartikel aus seiner Feder. Auch eine historische Herleitung des Verfahrens aus dem Jahre 1842 nimmt sich erneut diesem Streit an [100, 101].

Im Jahre 1837 führte Ivánchich in Pest erfolgreich die erste „blinde“ Lithotripsie aus. In Wien musste er 1838 nochmals durch das Rigorosum sein medizinisches Wissen (als gebürtiger Ungar) nachweisen, da für die Tätigkeit in Wien (also Österreich) nur Prüfungen an der Wiener Fakultät selber oder an der deutschen Universität Prag, in Innsbruck oder in Krakau anerkannt wurden und „Pesth“ als Ausland angesehen wurde [102–104]. Die Wiener Medizinische Fakultät war nach den Josephinischen Reformen die vorgesetzte Behörde für alle praktischen Ärzte und Wundärzte [105]. Für ein Rigorosum mussten nach der Ordnung von 1872 66 fl. (Gulden) ca. 666 € [106] gezahlt werden, die unter den prüfenden Ordinarien aufgeteilt wurden.

Zu diesem Zeitpunkt bedeute die „blinde“ Blasensteinlithotripsie nicht nur in Wien ein neues, innovatives und auch lukratives Betätigungsfeld, was die zeitgenössischen Quellen ausführlich belegen. Es diente weiterhin dem Wissensexport der Wiener Medizinischen Schule in andere Staaten [107–109].

Sofort begann von Ivánchich ein reiches publizistisches Schaffen, insbesondere in der *Wiener medizinische Wochenschrift* sowie der *Allgemeine Wiener medizinische Zeitung* zum Thema Blasensteinlithotripsie sowie zum benachbarten Gebiet der inneren Urethrotomie.

In späteren Jahren fasste von Ivánchich seine Publikationen in Sammelbänden zusammen und referiert hierzu auch auf der Naturforscherversammlung [110, 111]. Seine Zeitgenossen wiesen übereinstimmend darauf hin, dass er „Utraquist“ sei, also sowohl für den „offenen“ Steinschnitt wie die „blinde Lithotripsie“ einträte.

**Figure 5:**
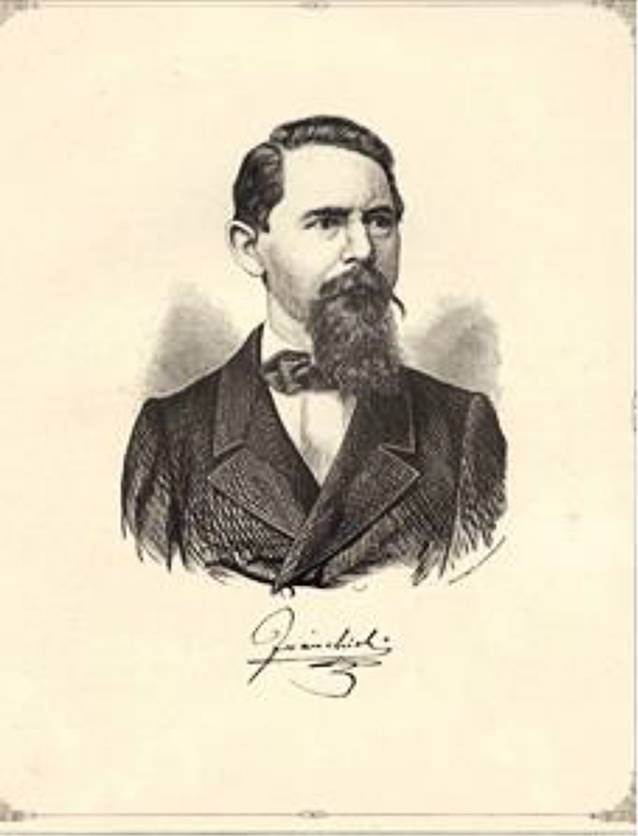

Diese Aussage wurde von seinen Zeitgenossen als wichtige Aussage weiter kolportiert, da Ivánchich so geschickt den kollegialen Konflikt, der seit Vinzenz von Kern um die Frage „offene Operation“ oder „transurethraler Eingriff“ besonders in Wien lebhaft geführt wurde, vermied. Durch seine hohen endourologischen Fallzahlen und seine Therapieerfolge behaupte er nicht nur seine wissenschaftliche Position, sondern konnte diese bedeutend ausbauen (Abb. 5).

## Die Diskussion um die Steintherapie in Wien um 1835

In Wien besaß seit Vinzenz von Kern (1760–1829; [112]) und seiner Publikation [113] die „offene“ Blasensteintherapie eine besondere Bedeutung, die neue Operationsmethode „blinde“ Lithotripsie wurde von der jüngeren Generation, von den Operateuren Joseph Wattmann Freiherr von Maëlcomp-Beaulieu (1789–1866) und Franz Schuh (1804–1865) vertreten, wobei Wattmann [114] noch ganz der primär von Jean Civiale [115–117] angegebenen Technik verhaftet blieb, wohingegen sich Victor von Ivánchich der Heurteloupschen Methode [118] zuwandte, die eine deutlich bessere Steindesintegration in einer Sitzung ermöglichte.

Ab 1842 bestand für Viktor von Ivánchich die Möglichkeit, seine Patienten am Allgemeinen Krankenhaus (AKH) in Wien behandeln zu können, da ihm ein Krankensaal „in der Mitte abgeteilt nach Männern und Weibern“ zugewiesen wurde, jedoch ohne Anspruch auf Bezahlung, was sich dann direkt in einer Publikation niederschlug [119, 120]. Das Allgemeine Krankenhaus in Wien unterstand dem Ministerium des Inneren, die Organisation der Universität und die Lehre unterstanden dem Ministerium für Unterricht [121]. Somit waren Professoren und Dozenten bei Personal- und Unterrichtsangelegenheiten wie die Assistenten dem Unterrichtsministerium diziplinarisch-organisatorisch unterstellt. Als Vorstand oder Mitarbeiter der Klinischen und „Reserveabteilungen“ waren diese der „Oberaufsicht des Spitals“ untergeordnet [122].

4. „… daß Dr. Ivanchich hierfür auch keine wie immer geartete Vergütung Anspruch mache, und daß dadurch dem Fonde keine neuen Ausgaben verursacht werden …“

5. „… daß diese Einrichtung von der Hand nur provisorisch und bis auf weitere Anordnung zu bestehen habe …“ [123].

Im Jahre 1844 finden wir ihn in Wien unter der Adresse Wollzeile 748 gemeldet [124].

Ab 1847 wandte Ivánchich die Äthernarkose an, die Franz von Schuh (1804–1865) in Wien am 27. Januar 1847 nach Tier- und Menschenversuchen erstmals demonstriert hatte [125, 126]. Er arbeitete deshalb mit dem Zahnarzt Joseph Weiger (1810–1863), der hierüber promoviert und publiziert hatte, eng zusammen [127–129]. Nach dem Tode Weigerts 1863 übernahm diese Position Lippmann Phillip Rabatz (1829–1890; [130]). Von Ivánchichs Vorliebe für die Äthernarkose wurde von den frühen Autoren nicht restlos geteilt. Johann Friedrich Diffenbach (1792–1847), Berlin, erhob hiergegen insofern Einwände, wenn er auch den allgemeinen Wert nicht in Abrede stellte, dass das Risiko einer Blasenwandverletzung unter Narkose deutlich höher sei, da der Patient normalerweise bei diesem Eingriff überhaupt keine Schmerzen haben sollte [131].

**Figure 6:**
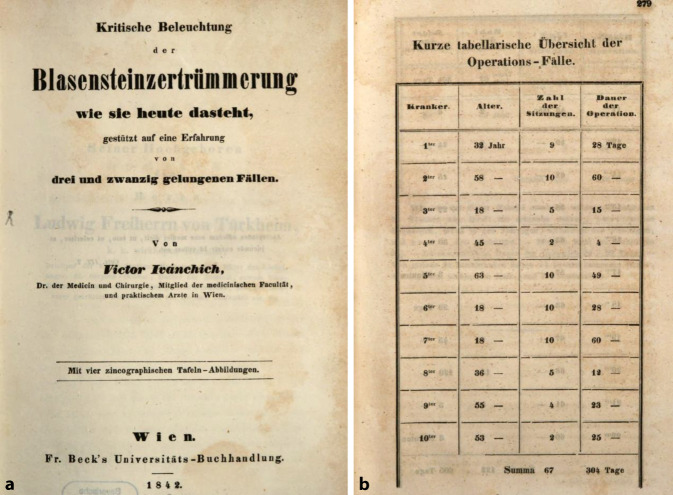

Von Ivánchichs Zeitgenossen wurde seine Publikation von 1842 „Kritische Beleuchtung der Blasensteinzertrümmerung, wie sie heute dasteht, gestützt auf eine Erfahrung von 23 Fällen“ stark beachtet und positiv kritisch rezensiert ([132]; Abb. 6 und 7).

… für Deutschland unerhört reicher Erfahrung auf dem betreffenden Gebiete abgefaßte Schrift, die sich die besten französischen lithotriptischen Werken (sit venia verbo!) an die Seite stellt … weil sie für keine der Ansichten Parthei nimmt … [133].

**Figure 7:**
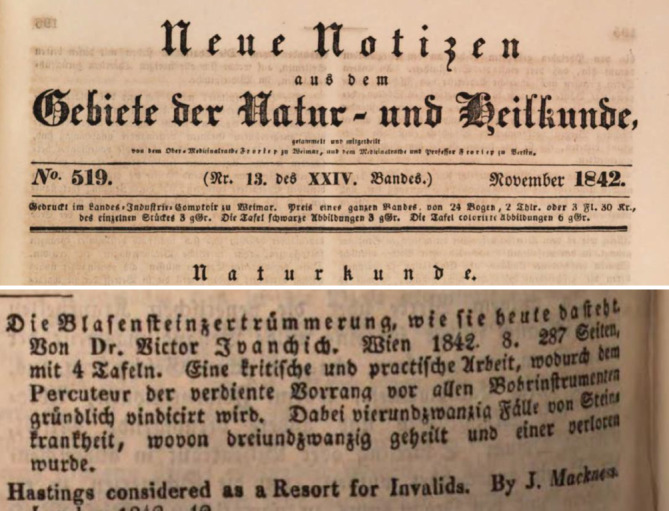

Trotz dieser Diskussionen setzte er seine statistischen Publikationen auf dem Gebiet der „blinden Blasensteinlithotripsie“ weiter fort, was schließlich zur Anerkennung der Methode führte [134–138].

## Therapie von Harnröhrenstrikturen – ein Betätigungsfeld der sich differenzierenden Urologie

Zusätzlich publizierte von Ivanchich ab 1846 zur Therapie von Harnröhrenstrikturen. Diese waren ein häufiges Krankheitsbild zu dieser Zeit infolge von gonorrhoischen Urethritiden, welche in der Mitte des 19. Jahrhunderts vor der Entwicklung eines differenzierten antiseptischen Therapieansatzes mit Spülungen nur unzureichend behandelbar waren [139, 140]. Bei der „inneren“ Urethrotomie, die sich neben der offen operativen herausgebildet hatte, waren im ersten Drittel des 19. Jahrhunderts zwei operative Varianten bekannt: zum einen die „retrograde“ Schlitzung, die beispielsweise Richard-Antony Stafford (1801–1854) oder auch Victor v. Ivánchich favorisierten und die „anterograde“ Urethrotomie nach Jules-Germain-François Maissnoeuve (1809–1894; [141]).

**Figure 8:**
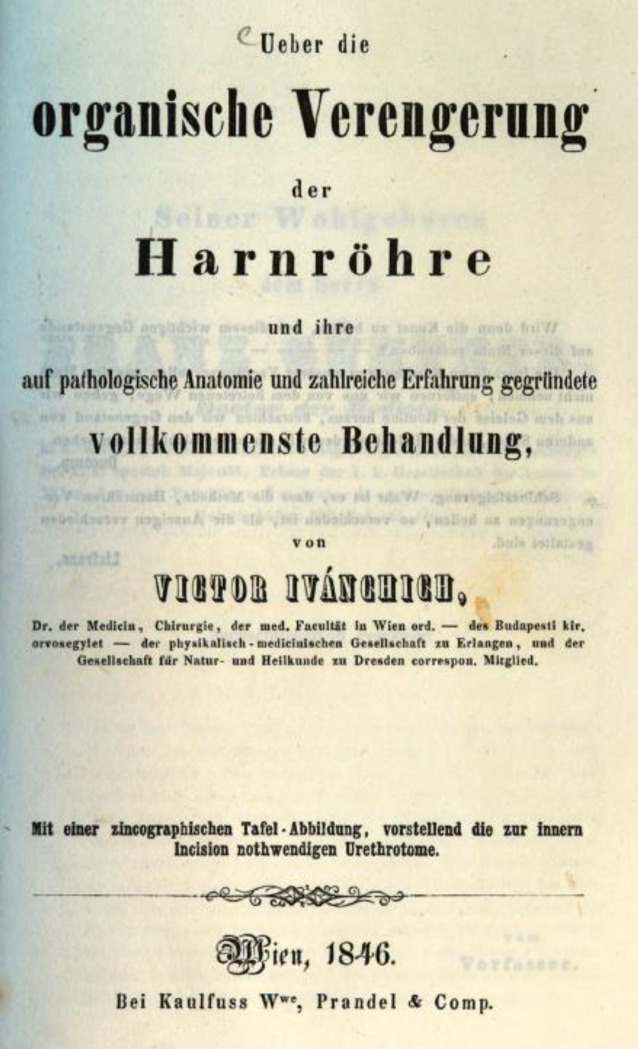

Durch Modifikation des „Scarificateurs de Ricord“, ein Modell hatte Ivánchich 1839 mit einer Sendung von Lithotriptoren von dem Instrumentenbauer Charrière in Paris erhalten [142], konnte er gute Erfolge erzielen, wie die Zeitgenossen feststellten, ohne jedoch auf publizistische Schwächen einer erfolgten Publikation ausführlich hinzuweisen (Abb. 8, 9 und 10).

… In Bezug auf das Historisch-Literarische könnte noch mancher Mangel an dem vorliegenden Buche aufgezeigt werden. – Die Form ist sehr vernachlässigt und der Styl zum Theil ungrammatisch und undeutsch, er ist mit vielen französischen Phrasen und mehrfachen Provincialismen vermischt. Dem slavischen Klang des Namens nach ist Herr Ivanchich vielleicht nicht in Deutschland geboren, in diesem Fall hätte er aber einem Deutschen das Buch zur Durchsicht übergeben können …[143].

**Figure 9:**
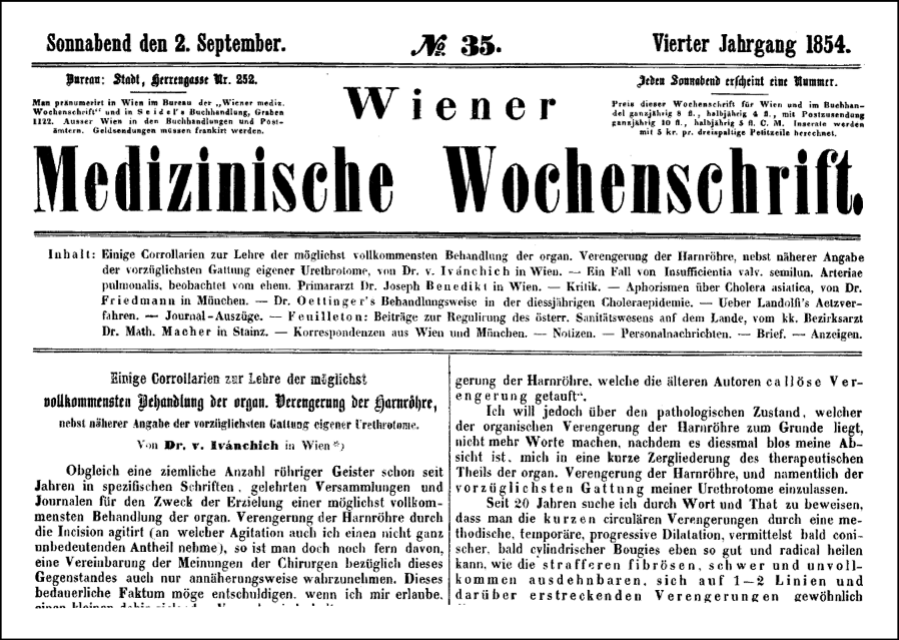

**Figure 10:**
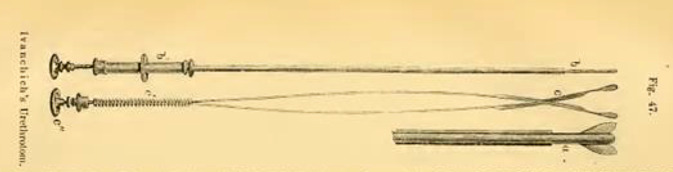

## Die Erteilung der „Venia legendi“ an Victor von Ivánchich 1851

Im Jahre 1851 erhielt Victor Ivánchich „nach Ansuchen“ die „Venia legendi“ der Medizinischen Fakultät Wien. Aufgrund des im Universitätsarchiv Wien erhalten gebliebenen Amtsvorgangs [144], lässt sich der Ablauf nachvollziehen. Ivánchich sandte ein ausführliches Schriftstück an die Fakultät, in dem er neben einem detaillierten Lebenslauf und Publikationen selbstsicher die Notwendigkeit für die Lehre der „Erkrankungen der Harnorgane“ auf mehreren Bögen detailliert darlegte und um eine Erteilung einer „Privatdozentur für Urologie“ „nachsucht“.

… dem Gehorsamst Gefertigten bleibt zu beweisen übrig:

I. daß die Privatdozentur der Urologie in wissenschaftlicher Beziehung nützlich sei und

II. daß dieselbe die Eigenschaften besitzet, welche der hohe ministerielle Erlaß von dem Privatdozenten im allgemein, die Lehre der Urologie aber speziell fordert.

Der zum Gutachter bestellte Chirurg Franz Schuh (1804–1865) fasste im Schriftstück vom 25.04.1851 zusammen:

„… Herr Victor von Ivanchich, praktischer Arzt in Wien, hat sich einen ehrenvollen Namen in Deutschland gemacht …“ .

… äußert sich Referent dafür, daß Dr. Ivanchichs Bitte um Verleihung einer Docentur XXX (ausgestrichen) für die Krankheiten der Harnorgane in allen Punkten bescheiden u. billig erscheine. Er trägt somit auf Verleihung der Docentur u. Befreiung nach Collegium, nicht aber auf Befreiung von der Probevorlesung an, da der letztere Akt ein öffentliche ist, u. dadurch nicht nur das Professoren Collegium sondern das ganze ärztliche Publicum als … auftrit(t) [145].

Es scheint über den Vorgang in der Fakultätssitzung keine besondere Debatte und Aussprache gegeben zu haben, jedenfalls wurde hierüber kein Eintrag gefertigt. Seinen Probevortrag am 3 Mai 1851 hielt Ivánchich „Ueber Krankheiten der Prostata“ [146].

Infolge der durch die 1848er-Revolution in Wien erstrittenen Lehrfreiheit und die sog. Thun’sche Universitätsreform [147, 148] sollte der Lehrkörper „vermehrt“ werden, was nach der Auffassung der Wiener Medizinhistorikerin Erna Lesky (1911–1986) den Beginn der akademischen Etablierung von Spezialfächern in Wien bedeutete [149]. Für das sich entwickelnde Fachgebiet der Urologie war durch die innovative, minimal-invasiven Behandlungsform der Lithotripsie von Harnsteinen sowie der „blinden Urethrotomie“ bereits vorher, 1842 am AKH Wien, Ivánchich ein Krankensaal zur Verfügung gestellt worden, initial noch ohne Lehrtätigkeit, nur zur Krankenversorgung.

**Figure 11:**
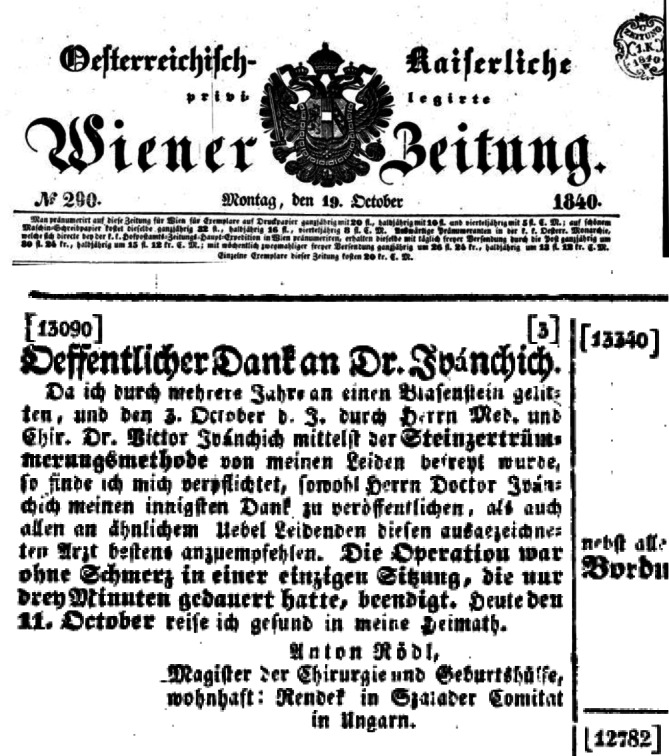

Wie Ivánchich seine Möglichkeit zur Lehre vor Studenten im Einzelnen genutzt hatte, konnte bisher anhand der spärlichen Quellen und Zeitzeugenhinweise noch nicht näher analysiert werden. Er war in der glücklichen Lage, durch die bereitgestellten Betten im AKH über Patienten zur Lehre problemlos verfügen zu können. Ivánchichs vielfältige Publikationen, die er seinen beiden Themen, die ihm am Herzen lagen wie „blinde Blasensteinlithotripsie“ sowie „innere Urethrotomie“, lassen indirekt auf sein persönliches Engagement schließen wie auch Hinweise seiner Patienten, die werbewirksam für ihn auftraten (Abb. 11).

Victor von Ivánchich erhielt 1872 von Russland den Stanislausorden, einen Orden, der von Zar Alexander I (1777–1825) als eigentlich polnischer Orden in das russische Regelement (ab 1831–1917) der Orden einverleibt worden war [150].

Im Jahre 1885 erhielt er in Österreich den „Orden der Eisernen Krone III. Klasse“, der besonders für das aufstrebende Bürgertum eine besondere sozialhistorische Bedeutung erlangte, da dieser für mehrfache namhafte Geldspenden vergeben wurde, die auch Ivánchich aus seinem Vermögen mehrfach getätigt hatte [151–153].

Weiterhin war er Mitglied bzw. korrespondierends Mitglied mehrerer lokaler wissenschaftlicher Vereinigungen wie der K. u. K. Gesellschaft der Ärzte in Wien [154], der Ärztevereinigung zu Dresden ab 1842 [155], zu Bern, der physikalisch medizinischen Gesellschaft zu Erlangen 1842 (korr.) [156], was ihm bei der Begutachtung durch die Fakultät zur Erlangung der „Venia legendi“ zum Vorteil gereicht hatte.

Diese Mitgliedschaften sind ein Indikator, dass er sich im Diskurs mit den Fachkollegen einen Namen erworben hatte und auch über persönliche Netzwerke verfügte, die es ihm erlaubten, positiv beurteilt zu werden, da zu dieser Zeit immer Bürgen für die Aufnahme in wissenschaftliche Fachgesellschaften, wie noch heute, erforderlich waren. Auch scheint er sich hier besonders nach der Bereitstellung von Betten bemüht zu haben, da eine zeitliche Koinzidenz der Verleihungen ab 1842 besonders auffällt.

Viktor von Ivanchich starb am 09. März 1892 und wurde auf dem Wiener Zentralfriedhof (Gruppe 41 A, Reihe 1, Nr. 10.) beigesetzt [157, 158].

**Figure 12:**
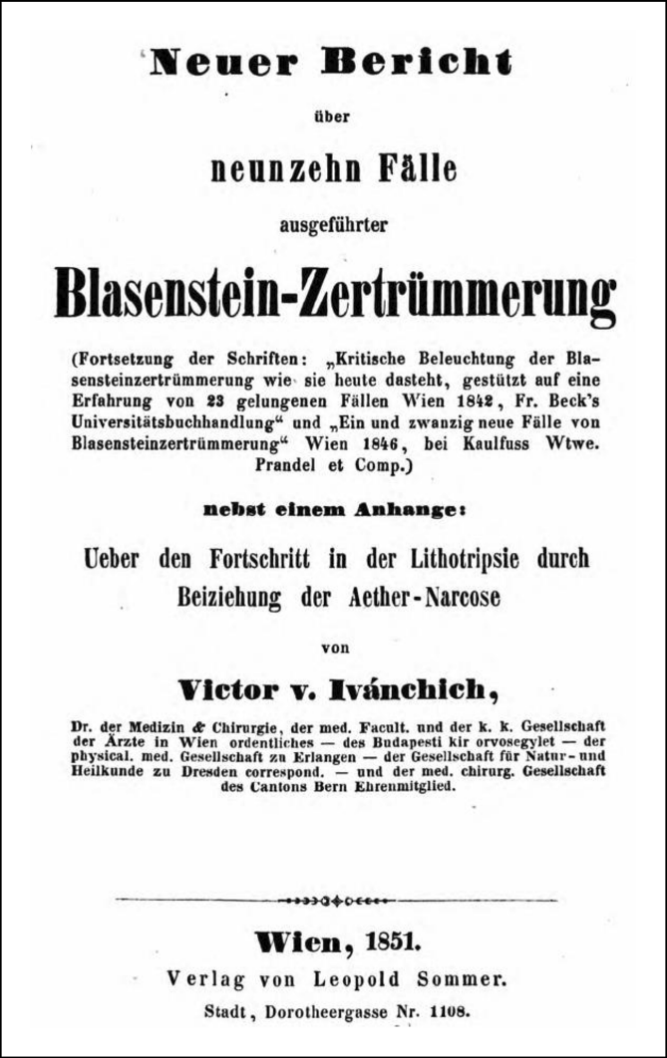

Victor von Ivánchich gelang es, unterstützt durch seine Position, die neuen endourologischen Methoden in einem breiten Rahmen zu publizieren, wobei er geschickt auch Mehrfachverwertungen seiner wissenschaftlichen Ergebnisse [159, 160] einsetzte, so zum einen als Einzelfallbericht, wie auch in Zusammenfassungen in Monographien. Weiterhin nutze er seine wissenschaftliche Dispute beispielweise über Harnröhrenstrikturen [161], die in den Journalen abgedruckt wurden, zur Verbreitung seiner Auffassungen (Abb. 12 und 13).

… Aus der vorliegenden Schrift wird der Leser ersehen, daß die Lithotripsie durch ihre Verbindung mit der Aether-Narkose, nun schon an extensiver Wirkung und Verbreitung, und an intensiver Vervollkommung eine Höhe erreicht hat, die wenig mehr zu wünschen übrig lässt so dass an dem Bürgerrecht (!) der Lithotripsie in der Chirurgie zu zweifen, heute schon Fevel an der Kunst genannt werden müsste ….. Vorwort S. III.–IV.

**Figure 13:**
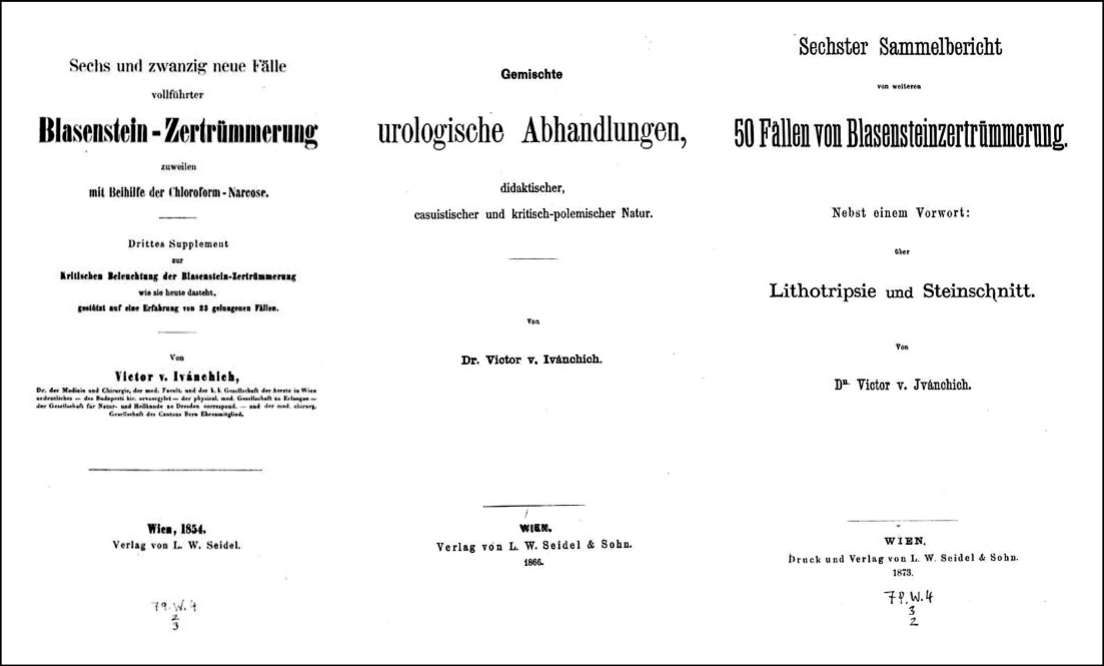

## Ergebnisse – Fazit für die Praxis

Habilitationen und die Erteilung der „Venia legendi“ in einem „neuen“, sich differenzierenden medizinischen Fachgebiet sind ein wichtiger Indikator der Fachdifferenzierung und Fachetablierung sowie ein Exzellenzkriterium im Kanon der universitären Fächer. Die Habilitanten sind von den Gutachtern benachbarter, etablierter Disziplinen abhängig. Denn diese neuen Disziplinen bedeuten für die etablierten Fächer neben einer wissenschaftlichen oft auch eine pekuniäre Konkurrenz, gerade in der operativen Medizin (Therapie von Blasensteinen: „offen“ – Lithotripsie, Behandlung des Prostataadenomes: Adenomektomie – TUR P).

Diese frühen Habilitationen zielen in Wien wie in Berlin besonders auf aktuelle neue technische, minimal-invasive Entwicklungen wie die „blinde“ Lithotripsie oder die Zystoskopie (Max Nitze) ab.

In Frankreich erhielt Jean Casimir Felix Guyon (1831–1920) 1877 eine volle „Professur für Chirurgische Pathologie“, nachdem er bereits 1867 Abteilungsvorstand am Hôpital Necker nach dem Tode Jean Civiales geworden war und seit 1863 als Agrégé (einer Habilitation vergleichbar) an der Universität Paris lehrte. Erst 1890 war er „Professor für die Klinik des Harnapparates“.

Mit der Habilitation und Erteilung der „Venia legendi“ war im deutschen Sprachraum meist nur eine lose Verbindung mit der Universität als Privatdozent verbunden. Dies bedeutete keine finanzielle Vergütung oder „Remuneration“ oder sonstige universitäre finanzielle Unterstützung. Die neuen Spezialfächer an den Universitäten führten meistens zunächst keine Betten an den Universitätskliniken oder Medizinischen Akademien; das bedeute, dass die Privatdozenten ihre eigenen Patienten für die Vorlesungen „mitbringen“ mussten bzw. die Kurse in ihrer Privatpraxis hielten [162]. Damit bildeten die „neuen Privatdozenten“ auch keine wirtschaftliche Konkurrenz zu den bereits etablierten Hochschullehrern. Von Ivánchich war hier in einer glücklichen Ausnahmesituation, da er schon vor der Erlangung der Venia legendi einen Krankensaal am AKH kostenlos betreuen durfte, was deutlich zu seiner Expertise beitrug, da er hier einfache Möglichkeiten der Patientenakquise und postoperative Behandlung besaß.

Die Universitäten sparten hierdurch eigenes, bezahltes ärztliches Personal für zu betreuende Krankenbetten und somit Unterhalts- und Personalkosten, profitierten aber gleichzeitig von jungen Forschern und Klinikern, deren neue Expertisen wesentlich zum universitären Renommee beitrugen. Gemeinsame Voraussetzung in Österreich-Ungarn sowie Preußen bzw. dem Deutschen Reich war die nachgewiesene, eigenständige klinische Erfahrung insbesondere in technisch geprägten, innovativen Bereichen (Lithotripsie, Zystoskopie), die gleichzeitig den Protagonisten aber chirurgischerseits dann auch wieder als fachliche Be- oder Einschränkung und zusätzliche Hyperspezialisierung, die nicht mehr das Gesamtfach im Blick habe, angelastet wurde^7^ [164].

Es fällt auf, dass durch die Berufung der unbesoldeten Privatdozenten den Hochschulen der Einfluss auf besonderes, „neues“ Patientengut und dessen Diagnostik und Therapie eröffnet wurde bzw. sich dieses durch Schaffung neuer Dozenturen erschloss. Gerade in sog. Ferienkursen demonstrierten diese habilitierten, unbezahlten Privatdozenten für ausländische Studenten besondere „Specialitäten“, die das Gesamtrenommee einer Hochschule deutlich steigerten [165]. In Wien wurde zur Beförderung des „Wissenschaftstourismus“ 1904 eine „American Medical Association (AMA) of Vienna“ gegründet, die das Kurswesen über Jahrzehnte organisierte [166–168]. Auch herrschte in Wien eine gewisse Liberalität bei der Vergabe der „Venia legendi“. Die jungen Dozenten mussten sich hier aber auch die Möglichkeiten der Lehre in Kooperation mit Leitern von Einrichtungen selber schaffen, denn eine Nichtausübung der Lehrtätigkeit über mehrere Semester hätte den Entzug der „Venia legendi“ hier zur Folge gehabt („Titellehre“). Dies war in Wien ein wichtiger Grund zur Errichtung der „Allgemeinen Wiener Poliklinik“ [169, 170] und dem sehr frühen Aufbau einer urologischen Abteilung durch Robert Ultzmann (1842–1889; [171]).

## Footnotes

1 Der Begriff des „langen“ 19. Jahrhunderts geht auf den britischen Historiker Eric Hobsbawm (1917–2012) und seiner gleichnamigen Publikation, die vor kurzem wiederaufgelegt wurde, zurück. (Das lange 19. Jahrhundert Europäische Revolutionen, Die Blütezeit des Kapital, Das imperiale Zeitalter. Theiss, Darmstadt), wurde auch von Jürgen Krocka für den 13. Band des seit 1891 erscheinenden Gebhardtschen Handbuches zur Geschichte verwandt [15].

2 NSDAP-Mitgliedsnummer: 1865995 (vgl. [35]).

3 Gustav Jurié Edler von Lavandal, 1842–1924, General-Chefarztes des Souv. Malteserordens, Hofrat, studierte in Wien, Promotion 1865, Habilitation 1874 Chirurgie der Harn- und Geschlechtswerkzeuge, 1887–1908 Primararzt, Spital der Barmherzigen Schwestern, Wien-Gumpendorf, daneben Privatpraxis, die vom Adel gern aufgesucht wurde, Gründung des Rudolfinervereins 1875 [53].

4 Gustav Juriés Habilitationsansuchen ist leider im Universitätsarchiv in Wien nicht erhalten geblieben, der Vorgang lässt sich aber rekonstruieren. Das Gesuch wurde am 22.11.1873 behandelt, Gutachter waren Dumreicher, Dittel, Billroth, Thema des Probevortrages vom 07.02.1874: „Katheterismus bei Hypertrophie der Prostata“ (MED S 52.9.5), Schreiben von U. Denk, Archiv Universität Wien vom 07.08.2018.

5 Erna Lesky erwähnt bei Ivnachich keine Habilitation, obwohl sie die Arbeit von Keller [54] in ihrem Werk „Die Wiener Medizinische Schule“ [55], zitiert.

6 Habilitation für Chirurgie 1938 (Tit. a. o. Professor; Krankenhaus Lainz, nach dem „Anschluss“ 1939 seiner Funktion als Primarius enthoben, Entzug der Venia legendi 1940, in der NS-Zeit als Militärarzt tätig).

7 Siehe [163] „… Wir erfahren nicht, wo und wie er sich geschult hat und ob wir ihm die Befähigung zutrauen dürfen, mehr noch zu können als ein Endoskop zu konstruieren und mit demselben die Krankheiten der Blase zu erkennen. In der Hoffnung, daß er durch seine Leistung auf einem Theile des betreffenden Gebietes auch in den Stand gesetzt wird, die anderen sich zu eigen zu machen, empfehle ich ihn für die Zulassung zur Habilitation als Privatdozent für Chirurgie …“, Ernst von Bergmann 07.11.1889, „Gutachten zur Habilitation“ Nitzes „Begutachtung des Nitzeschen Buches Lehrbuch der Kystoskopie“.
